# AbobotulinumtoxinA: A New Therapy for Hip Osteoarthritis. A Prospective Randomized Double-Blind Multicenter Study

**DOI:** 10.3390/toxins10110448

**Published:** 2018-10-31

**Authors:** Roberto Eleopra, Sara Rinaldo, Christian Lettieri, Andrea Santamato, Paolo Bortolotti, Carmelo Lentino, Carmine Tamborino, Araldo Causero, Grazia Devigili

**Affiliations:** 1 Neurological Unit 1, Fondazione I.R.C.C.S. Istituto Neurologico Carlo Besta, 20133 Milan, Italy; sara.rinaldo@istituto-besta.it (S.R.); grazia.devigili@istituto-besta.it (G.D.); 2 Neurological Unit, “S.Maria della Misericordia” University Hospital, 33010 Udine, Italy; christian.lettieri@asuiud.sanita.fvg.it; 3 Physical Medicine and Rehabilitation Unit, “Riuniti” Hospital, 71122 Foggia, Italy; andrea.santamato@unifg.it; 4 Neurological Unit, “Villa Rosa” Rehabilitation Hospital (APSS TN), 38057 Trento, Italy; paolo.bortolotti.tn@gmail.com; 5 Physical Medicine and Rehabilitation Unit, “Santa Corona” Hospital, (Local Health Agency 2 Savonese), 17027 Pietra Ligure (SV), Italy; carlenti.cl@gmail.com; 6 Neurological Unit, “Dell’Angelo” Hospital, 30174 Venice, Italy; carmine.tamborino@aulss3.veneto.it; 7 Institute of Orthopedic Surgery, “S.Maria della Misericordia” University Hospital of Udine, 33010 Udine, Italy; araldo.causero@asuiud.sanita.fvg.it

**Keywords:** botulinum toxin, hip, osteoarthritis, rehabilitation, pain

## Abstract

Hip Osteoarthritis (OA) causes pain and disability. Here we evaluate abobotulinumtoxinA (Dysport^®^) (AboBoNT-A) injections versus placebo as a novel treatment option to improve hip range of motion, pain and quality of life. This prospective randomized double-blind multicenter study (EudraCT # 2012-004890-25) recruited 46 outpatients with hip OA who were randomized 2:1 to the Treatment Group (TG; 31 subjects), or the Placebo Group (PG; 15 subjects). The TG received 400 U of AboBoNT-A injected into the adductor muscles, and the PG received placebo solution. The primary endpoints were the difference in Harris Hip Score (HHS) and Visual Analogic Scale for pain (VAS) at Week 4 between groups (TG vs. PG). Secondary endpoints were the change from baseline in HHS, VAS pain, Medical Research Council scale for muscle strength (MRC) and Short Form scale (SF-36) scores. In TG at Week 4, the HHS and VAS score were significantly improved compared to PG, and pairwise assessments showed significant improvements in HSS and VAS pain at each time point compared to baseline for TG. No significant changes were observed in MRC and SF-36 over time, though SF-36 showed a positive trend. There were no significant differences from baseline in the PG. No adverse events were detected in either treatment group. AboBoNT-A injections in hip OA improve range of motion and pain without any significant side effects.

## 1. Introduction

Hip Osteoarthritis (OA) is a major cause of pain and disability in Western populations [1]. Although hip OA most frequently occurs in the elderly, many cases can occur before retirement, when hip OA can seriously impact a patient’s ability to work [1]. As longevity increases and raised retirement ages require people to work longer, the scale of this problem is expected to grow [2,3,4]. Therefore, a recent study of Castell and coll. (2018) reveals that in Europe the incidence of hip OA is variable in the elderly, and higher in Italy (13.8%) in comparison to the overall Europe frequency (5.9%) [5].

Clinically, all patients experience the same impairment with hip OA: Diminution of range of motion, pain and functional incapacity, as well as altered biomechanics of walking. Severe hip OA is often treated with surgery, the main surgical technique being total hip arthroplasty (THA) [6,7,8]. Since 1950, surgical treatment has undergone rapid and remarkable development by means of THA [9]. Nevertheless, the failure of THA or the recuperation following surgery is sometimes difficult, in particular in elderly patients [10,11,12]. It is therefore useful to delay THA surgery, whilst guaranteeing a good quality of life to patients waiting for the operation (absence of pain and a wide range of motion).

Systematic reviews suggest that conservative treatment is effective and advisable in mild-to-moderate cases [13,14]. Conservative therapy includes both pharmacological and non-pharmacological treatments, aimed to decrease the mechanical stress across the joint and increase the stress levels that the joint can withstand [14]. Depending on the degree of damage to the hip joint, therapeutic options are: Drug therapy (NSAIDs, analgesics, hormones), anaesthetic block, injection of medications into the joint cavity, manual therapy (muscle and joint technology), physiotherapy and surgery [13,14].

In patients with hip OA, pain may be a consequence of prolonged or excessive contraction of the adductor muscles and Botulinum NeuroToxin serotype A (BoNT-A) could be proposed as an effective treatment [15]. It is known that BoNT-A is used for treatment of spasticity of the hip adductors and injections of BoNT-A have been used as an adjunctive measure to prevent or delay lateral migration of the femoral head in children with cerebral palsy [16,17]. It is hypothesized that BoNT-A injection into selected muscles involved in hip movements will reduce the pressure transferred by the femoral head against the acetabulum, as is achieved with the Voss operation [18,19]. This reduction in pressure aims to break, in an efficient and lasting manner if periodically treated, the vicious cycle induced by the combination of pain–contraction and contraction–pain. 

In our preliminary open-label non-controlled, multicenter study (Marchini et al., 2010) [20] we assessed the effectiveness of abobotulinumtoxinA (AboBoNT-A) injections in a small population of patients with hip OA. Patients were injected with aboBoNT-A into the adductor longus and magnus muscles of the thigh obtaining a significant reduction of hip pain.

The aim of this current study is to perform a prospective randomized double-blind multicenter study in a larger population of patients with hip OA to confirm our previous findings regarding the efficacy and clinical benefit of aboBoNT-A injections to improve hip range of motion and pain. 

## 2. Results

46 outpatients with hip OA were recruited and randomized 2:1 to either the Treatment Group (TG; 31 patients) or the Placebo Group (PG; 15 patients). At the study’s conclusion, a posteriori power analysis for the main outcomes (HSS, VAS and MRC) with an effect size of 1.0, accounting for a large effect, and an alpha level set at 0.05 express a power of 0.87.

At baseline, the TG included 16 females and 15 males between 28 and 81 years of age (mean age 62.2 ± 13.7 years) and the PG included 9 females and 6 males between 37 and 73 years of age (mean age 63.1 ± 10.6 years). The groups were balanced for age (*p* = 0.879) and sex (*p* = 0.139). At baseline, for the PG and TG, the mean weight was 74.0 ± 12.1 kg and 75.9 ± 14.8 kg, and the height were 1.64 ± 0.09 mt. and 1.69 ± 0.10 mt., respectively. The differences between the groups were not significant in both parameters (*p* = 0.163, at least).

**Figure 1:**
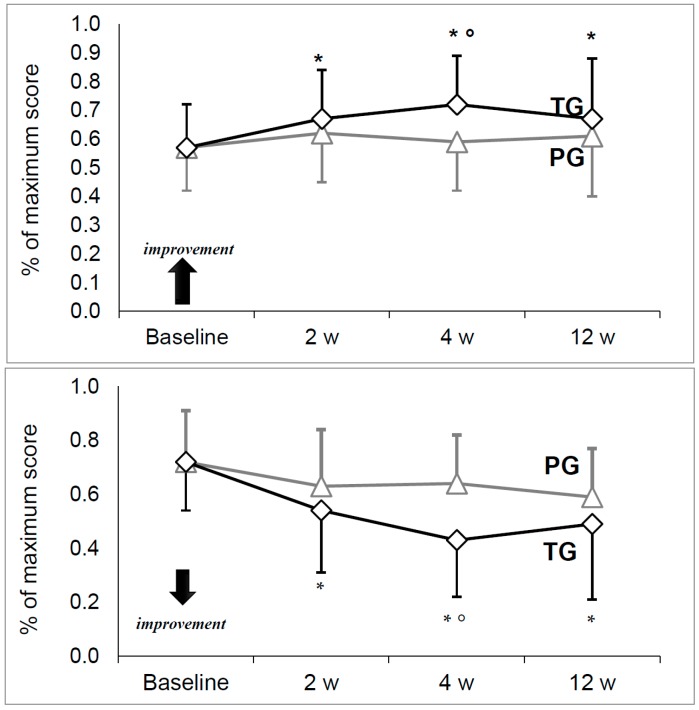
The HHS (upper) and VAS (lower) values over time in the Groups. Data are calculated as percentage of maximum score (100) and presented as mean ± standard deviation. Placebo Group (PG), *n* = 15 subjects and Treated Group (DG), *n* = 31 subjects. Statistically significant difference with the corresponding baseline value (*) or with the corresponding Placebo group value (°).

The Kellgren-Lawrence (KL) grade scores at baseline were 2.0 (2.0;3.0) and 3.0 (2.0;3.0) with no significant difference between the groups (*p* = 0.715). Scores at baseline and at each timepoint on the Harris Hip Score (HHS), Visual Analogic Scale for pain (VAS), Medical Research Council scale for muscle strength (MRC) and Short Form scale (SF-36, both physical and mental components) are summarized in Table 1 and Figure 1.

HHS and VAS were similar between treatment groups at baseline with no significant differences (Figure 1). Both HHS and VAS were stable in the PG, with no significant change between time points. On the contrary, a significant increase for HHS and decrease for VAS were observed in the TG (*p* = 0.000).

A pairwise comparison for the TG showed significantly greater HHS and lower VAS at each time point compared to the baseline value. As for comparisons between the groups, both HHS and VAS were significantly improved in the TG at four weeks (respectively: *p* = 0.026 and *p* = 0.001), though not statistically significant at other time points.

In the MRC and SF-36 (both physical and mental components) no significant changes were observed over time in either group (Table 1). Between groups, there were no significant differences, even though a slight positive trend (improvement) was observed in SF-36 scores over time, particularly the physical components at week twelve (*p* = 0.05).

We did not detect any statistical variation in systolic and diastolic blood pressure, body temperature and FC b/m (see Table 2).

None of the subjects examined had any major or significant adverse effect after treatment. In the TG, only two subjects had a transient (resolving in 24 h) knee-pain in the contralateral lower limb (untreated), unrelated to the study treatment. No important harm or unintended effects have been noticed in either group.

## 3. Discussion

Hip OA is a non-inflammatory disease consisting of degeneration of articular cartilage followed by degradation of joint surfaces that leads to the deformation and stiffness of the hip joint [21,22]. A dominant symptom of hip OA is pain on weight bearing or motion. 

In the last forty years the surgical treatment of hip OA has rapidly and remarkably developed by means of THA [6] but the revision surgery is sometimes complicated, especially in elderly people. Before implantation of THA, hip OA was commonly approached by surgical detachment of the most important muscles of the hip joint contracted, thus achieving the purpose of interrupting, in an efficient and lasting manner, the vicious cycle induced by the combination of pain-contraction and contraction-pain. 

In 1955 Voss [18,19] described a surgical procedure for the treatment of hip osteoarthritis, the so-called *temporary hanging hip*, entirely based on a new principle. In his opinion, the most important factor was that the muscles around the joint were in a state of increased tension; for this reason, the articular cartilage is put under abnormal pressure causing a so-called *osteoarthrosis pressure*. Accordingly, the therapeutic principle should be an attempt to break this condition of abnormal muscular tension. So, a neuromuscular blockage of these muscles could result in clinical benefit.

AboBoNT-A is a neurotoxin produced by the bacterium Clostridium Botulinum [23,24] and it is useful to treat focal dystonia and spasticity [25]. The clinical efficacy of aboBoNT-A depends on the dose administered and the site of injection. Most of the clinical effects are a consequence of the pharmacological action of the toxin on the cholinergic nerve terminals ending, causing the weakness of the muscles adjacent to the site of injection [26]. With therapeutic aboBoNT-A injections, the side effects are often transient and rarely severe, while the clinical benefit is usually observed over a period of 3–4 months with a maximal benefit after 4–6 weeks. The aboBoNT-A effect remains local and non-systemic significant effects are described. Injections can be repeated in time and this therapy appears useful over a long period [27].

Marchini et al. in 2010 reported an improvement in range of motion and pain by evaluating the HHS and VAS pain Score in 39 subjects affected by hip OA treated with aboBoNT-A injections, indicating that this might be an innovative, less invasive treatment with remarkable effects on clinical management, but the study protocol was unblinded and the study open-label [20]. In our prospective randomized double-blind multicenter study, we aimed to confirm whether administration of aboBoNT-A versus placebo on hip range of motion, pain and quality of life have an efficacy in subjects with hip OA.

Our results confirm the clinical improvement in joint mobility (HHS) and pain reduction (VAS) recorded at week four after aboBoNT-A injections compared to placebo, when a maximal peak aboBoNT-A effect is expected. Moreover, in the treated subjects (TG) but not in the placebo group (PG) a statistical difference for HHS and VAS has always been detected at each control visit in comparison to the baseline values, confirming the validity of this new treatment.

A similar positive trend of improvement in quality of life (SF-36) has been noticed in TG but without statistical significance (*p* 0.05 at week 12) while these results have not been observed in the PG. A possible explanation is that in our protocol subjects did not receive physical therapy after injection. It is probable that concomitant and dedicated physical treatment could result in a major benefit for the treated subjects, in whom hip pain is diminished and range of motion improvement. Eventually, a future dedicated randomized clinical trial could better clarify this.

Finally, there were some limitations to this study. Firstly, the small population recruited could influence the SF-36 statistical results among the TG and PG groups, even if a posteriori power analysis of our samples size found a power of 0.87, considering an alpha level set at 0.05, for the main outcomes (HSS, VAS, SF-36 and MRC).

Furthermore, the aboBoNT-A dosage used (400 U) could limit a more evident improvement in the TG if they were injected at higher dosage. We believe that future clinical trials could reasonably inject aboBoNT-A at a higher dose in consideration of the absence of any side collateral effects on muscular strength detected in our study. This could determine a possible long-lasting improvement in range of motion of hip (HHS) and significantly enhance quality of life (SF-36).

## 4. Conclusions

Our study confirms that aboBoNT-A treatment is an innovative well tolerated treatment in patients affected by hip OA and that injections could be repeated without any significant adverse effects. Therefore, we confirm the relevance of this new therapy in the orthopaedic or rehabilitation field, in particular when a THA surgery is contraindicated or delayed. The novelty of this treatment is that a single treatment with BoNT-A injected in the adductor muscles improves both joint range of motion and pain.

## 5. Materials and Methods

### 5.1. Study Design

We conduct a prospective investigator-initiated randomized double-blind placebo-controlled multicenter study (parallel groups) for hip OA aimed to evaluate the effect of injection of Abo-BoNT versus placebo on hip range of motion, pain and quality of life (EudraCT #2012-004890-25). The allocation ratio has been random and done at the coordinator center of Udine in a blind manner.

**Figure 2:**
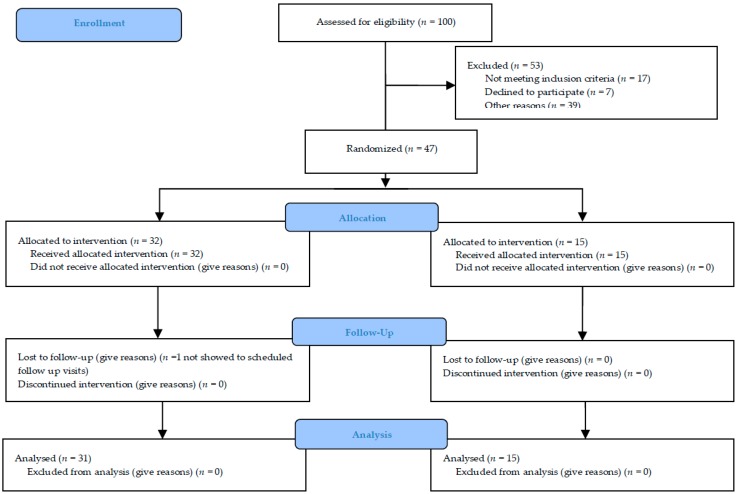
Flow-Chart Diagram of the Study.

The trial was originally intended for a sample size of about 100 subjects to evaluate and enrol in ten different centers, in order to have three different populations: The “safety population” (patients who receive at least one dose of study substance), the “intention-to-treat population” (patients who receive at least one dose of study medication and who have at least one primary efficacy evaluation after baseline visit) (ITT) and the “per protocol population” (patients in the ITT Population who do not violate any major entry condition and do not violate the protocol between inclusion and study completion) (PP). The duration of the study was nine months: Six months to enroll the patients and three months for the follow-up. The maximum duration of the study for each subject was 12 weeks and at the end of the study, 46/47 patients were included in the PP with a posteriori power study analysis of 0.87. The experimental protocol was approved by our Ethical Committee (Approval number #n. 1071 of 28 December 2012) and under Italian Health Authority (EudraCT # 2012-004890-25). Figure 2 summarized the study recruitment.

### 5.2. Study Participants

100 consecutive outpatients were evaluated during visits to the orthopedic departments because they were affected by primary or secondary symptomatic and painful osteoarthritis. 53/100 were excluded because they did not meet inclusion criteria or were not interested in participating in the study or for other reasons (e.g., far from hospitals). The patients subsequently enrolled were evaluated by the neurological or rehabilitation specialist for a possible BoNT-A treatment. They were enrolled in 5 different Italian hospitals (neurological or rehabilitation units): Udine, Venice-Mestre, Pergine-Trento, Pietra Ligure-Savona and Foggia. The other centers did not enroll any patients during the recruitment phase.

Subjects with primary or secondary symptomatic painful osteoarthritis were selected because of the presence of radiographic signs of hip OA by X-ray, with quantitative measurement of joint space width and determination of OA severity (grade 3–4 at the Kellgren-Lawrence (KL grading score) radiological grading scale [28]. Some of them performed also MRI more informative for the articolar cartilagine loss, bone marrow lesion and other connective and bone tissue abnormality [29].

Inclusion criteria were: age between 18 and 80 years; expected waiting time more of six months for surgical treatment from orthopaedic visit; Harris Hip Score (HHS) > 20 [30] and VAS pain score > 4 (minimal value defined as “significant pain”) [31].

Exclusion criteria were: Documented systemic allergy, pregnancy or women who were planning a pregnancy within six months from the treatment; previous treatment with BoNT-A with any kind of toxins, whatever the indication; physiotherapy or articular injections with analgesic, hyaluronic acid or corticosteroids in the previous 60 days from recruitment; history of relevant central or peripheral neurological diseases, patients who underwent a previous hip arthroplasty; subjects unable and/or unwilling to fully comply the study protocol; apparent remission of osteoarthritis within 3 months from the enrolment or subjects who brought changes on analgesic oral therapy in the previous 4 weeks.

All the voluntary subjects entered the study and gave their written voluntary consent, and all participants were told about the goal of the study, possible adverse reactions and about the treating physician in case of any complications.

### 5.3. Study Protocol (Interventions)

The eligible patients were randomly assigned (2:1) into two groups: Treated Group (TG) or Placebo Group (PG) according to a randomized plan. The choice of 2:1 proportion (TG or PG) has been decided before the beginning of the study in order to have more subjects in TG to evaluate the Abo-BoNT safety.

In TG, a total of 400 U of Abo-BoNT were injected using a dilution of a Dysport^®^ vial (500 U) into 2 mL of physiological solution to a concentration of 250 U/mL. Each subject received 250 U (1.0 mL of volume solution) in the adductor longus muscle and 150 U (0.6 mL of volume solution) in the adductor magnus muscle of the thigh of the hip OA affected side. In each muscle, we slowly injected 2 different muscular sites under electromyography guide-assistance. The patients of PG received 1.6 mL of physiological solution (placebo) as described above.

The subjects were examined during the recruitment visit (baseline, V0) and after injections at two (V1), four (V2) and twelve (V3) weeks post treatment. During each visit, all subjects were assessed with the HHS to test hip range of motion [30] the VAS for pain score [31], the MRC scale to assess muscular strength [32] and the SF-36 scale [33] to assess quality of life. The intensity of pain was assessed by the generic one-dimensional pain questionnaire VAS using a 10 cm length line from 0 (no pain) to 10 cm (the worst imaginable pain) [32]. Using a ruler, the score has been determined by measuring the distance between the “no pain” and the patient’s mark. In the presence of bilateral hip OA, only the most symptomatic side was treated.

### 5.4. Outcomes

The primary efficacy variable that has been considered is the HHS and VAS at week four between the two groups (TG vs. PG). Secondary efficacy variables are HHS, VAS, MRC and the SF-36 at each follow-up visit in comparison to baseline for each group. At week two, four and twelve, safety was assessed through the collection of adverse events (AEs), clinical and biochemical evaluations such as electrocardiogram, electrolytes, haematology and serum chemistry parameters, urine tests and changes in vital signs. All those tests were required by the Italian national agency (AIFA). No change was made to trial outcomes after the trial commenced.

### 5.5. Randomization

We used a centralized randomized plan with dedicated handmade PC software located at the coordinator center of Udine. A list of randomizations was created using randomized permutation blocks within layers, thus considering the centers involved and balancing the number of subjects per center. Each patient was matched with a randomization code that will also correspond to the patient’s identification in the study. At V0, the operator (blinded) who prepared the solution contacted by a dedicated phone line the coordinator center that allocate the patient in TG or PG group.

### 5.6. Blinding

A blinded examiner (neurologist or rehabilitator) examined the subjects during the recruitment visit (V0) and after Abo-BoNT injections, at V1, V2 and V3. The treatments were performed in a blinded manner: In each center, one investigator prepared the solution (drug or placebo), while a second investigator (blinded) managed and performed the injections. Only the operator who prepared the solution knew what kind of substance was injected: Abo-BoNT or Placebo (saline). The Investigator responsible for preparing the solution was never involved directly in the injections or in the follow-up and he communicated the identity of the treated and untreated subjects to the Principal Investigator of the coordinator center. The other investigator (blinded) collected the clinical data (CRF) and the Abo-BoNT side adverse effects.

### 5.7. Statistical Analysis

Each continuous data set was tested for the normality of the data by means of the Shapiro-Wilk test and by Q-Q normality plots. Equality of variance was also tested by the means of the Levene test and Q-Q normality plots of the residuals. Most of the continuous datasets were treated as ordinal data by non-parametric tests, due to the failure to meet the required assumption for using parametric analyses seen [34,35]. Data are presented as median (25th; 75th percentile) or mean ± standard deviation.

The balancing of the groups by age and sex were tested by the Mann-Whitney U-test and Fisher exact test, respectively. The significance of the differences in weight and height between the groups at baseline was evaluated through an unpaired sample *t*-test; while the significance of the differences in KL grade evaluated through the Mann-Whitney U-test. For each other parameter, the Mann-Whitney U-test was used to assess the significance of differences between the groups within each time point. The significance of the differences over time within either group was assessed using a Friedman test followed by a Bonferroni-corrected Wilcoxon paired sign rank test, when appropriate. The significance of the difference in the distribution of the different ECG patterns (normal, pathological and unspecific) between the groups at baseline was assessed by a chi-squared test.

Sample size was calculated assuming a common standard deviation of 24 and considering the relationship between subjects in the TG and PG will be equal to 2:1. Power was estimated at 90% to highlight an improvement of the HHS score in TG vs. PG of 18 points. The test was selected in two queues with a level of significance of 0.05.

## Figures and Tables

**Table 1:** The HHS, VAS score, MRC and SF-36 values over time in the Groups.

Parameter	Group	Time Point				
		Baseline	2 w	4 w	12 w	Diff. for Each Group
**HHS**	**PG**	60 (45;66)	65 (46;69)	66 (46;67)	62 (48;66)	**NS**
	**TG**	56 (44;67)	62 (56;78) *	74 (60;86) *	66 (53;87) *	***p* = 0.000**
	**Diff. between groups**	**NS**	**NS**	***p* = 0.026**	**NS**	
**VAS**	**PG**	7.2 ± 1.9	6.3 ± 2.1	6.4 ± 1.8	5.9 ± 1.8	**NS**
	**TG**	7.2 ± 1.8	5.4 ± 2.3 *	4.3 ± 2.1 *	4.9 ± 2.8 *	***p* = 0.000**
	**Diff. between groups**	**NS**	**NS**	***p* = 0.001**	**NS**	
**MRC**	**PG**	5 (4;5)	5 (4;5)	5 (5;5)	5 (5;5)	**NS**
	**TG**	5 (5;5)	5 (5;5)	5 (5;5)	5 (5;5)	**NS**
	**Diff. between groups**	**NS**	**NS**	**NS**	**NS**	
**SF-36 (PC)**	**PG**	34.0 (31.1;38.7)	34.3 (28;42.3)	33.6 (29.0;42.3)	34.4 (25.3;42.7)	**NS**
	**TG**	30.3 (22.2;38.1)	32.4 (28;40.0)	35.0 (26.9;47.2)	36.8 (30.4;43.0)	**NS**
	**Diff. between groups**	**NS**	**NS**	**NS**	**NS**	
**SF-36 (MC)**	**PG**	40.2 (36.8;48.1)	43.4 (37.7;49.0)	39.0 (35.0;49.3)	47.0 (36.7;54.0)	**NS**
	**TG**	45.2 (36.0;55.9)	47.1 (34.7;54.1)	42.2 (33.8;54)	49.3 (38.0;55.5)	**NS**
	**Diff. between groups**	**NS**	**NS**	**NS**	**NS**	

Data are presented as median (25th; 75th percentiles) or mean ± standard deviation. Placebo Group (PG), *n* = 15 and Treated Group (TG), *n* = 31. Diff. = significance of the difference “between groups” and for “each group” (in comparison to baseline) over time. * = statistically significant difference with the corresponding baseline value (*p* < 0.05, at least). NS = difference not statistically significant.

**Table 2:** The systolic and diastolic blood pressure, body temperature and HR values over time in the Groups.

Parameter	Group	Time Point				
		Baseline	2 w	4 w	12 w	Diff. for Each Group
**Systolic BP** (mm Hg)	PG	126.3 ± 10.1	129.3 ± 11.5	130.0 ± 13.6	133.7 ± 13.7	**NS**
	TG	131.9 ± 14.6	131.0 ± 13.4	132.3 ± 12.3	130.5 ± 13.2	**NS**
	**Diff. between group**	**NS**	**NS**	**NS**	**NS**	
**Diastolic BP** (mm Hg)	PG	72.0 ± 9.2	75.7 ± 11.5	72.7 ± 8.6	73.3 ± 8.8	**NS**
	TG	76.0 ± 9.2	77.4 ± 10.8	75.7 ± 9.9	74.8 ± 8.6	**NS**
	**Diff. between group**	**NS**	**NS**	**NS**	**NS**	
**Body Temp** (°C)	PG	36.2 ± 0.3	36.1 ± 0.2	36.2 ± 0.3	36.2 ± 0.3	**NS**
	TG	36.1 ± 0.3	36.1 ± 0.2	36.0 ± 0.3	36.1 ± 0.3	**NS**
	**Diff. between group**	**NS**	**NS**	**NS**	**NS**	
**HR** (b/m)	PG	69.1 ± 8.9	68.3 ± 6.2	67.2 ± 6.1	67.5 ± 5.6	**NS**
	TG	67.5 ± 7.2	69.3 ± 8.7	67.6 ± 7.9	68.2 ± 8.8	**NS**
	**Diff. between group**	**NS**	**NS**	**NS**	**NS**	

Data are presented as mean ± standard deviation. Placebo Group (PG), *n* = 15 and Treated Group (TG), *n* = 31. NS = difference not statistically significant between the groups within each time point, or over time within either group.

## References

[1] Zambon S., Siviero P., Denkinger M., Limongi F., Victoria Castell M., Van der Pas S., Otero Á., Edwards MH., Peter R., Pedersen N.L. (2016). Role of Osteoarthritis, Comorbidity, and Pain in Determining Functional Limitations in Older Populations: European Project on Osteoarthritis. Arthritis Care Res..

[2] Cross M., Smith E., Hoy D., Nolte S., Ackerman I., Fransen M., Bridgett L., Williams S., Guillemin F., Hill C.L. (2014). The global burden of hip and knee osteoarthritis: Estimates from the Global Burden of Disease 2010 study. Ann. Rheum. Dis..

[3] Harris C.E., Coggon D. (2015). Hip Osteoarthritis and Work. Best Pract. Res. Clin. Rheumatol..

[4] Yoshimura N., Muraki S., Nakamura K., Tanaka S. (2017). Epidemiology of the locomotive syndrome: The research on osteoarthritis/osteoporosis against disability study 2005–2015. Mod. Rheumatol..

[5] Castell M.V., van der Pas S., Otero A., Siviero P., Dennison E., Denkinger M. (2015). Osteoarthritis and frailty in elderly individuals across six European countries: Results from the European Project on OSteoArthritis (EPOSA). BMC Musculoskelet. Disord..

[6] Gademan M.G., Hofstede S.N., Vliet Vlieland T.P., Nelissen R.G., Marang-van de Mheen P.J. (2016). Indication criteria for total hip or knee arthroplasty in osteoarthritis: A state-of-the-science overview. BMC Musculoskelet. Disord..

[7] Rolfson O., Bohm E., Franklin P., Lyman S., Denissen G., Dawson J., Dunn J., Chenok K.E., Dunbar M., Overgaard S. (2016). Patient-reported outcome measures in arthroplasty registries. Report of the Patient-Reported Outcome Measures Working Group of the International Society of Arthroplasty Registries Part II. Recommendations for selection, administration, and analysis. Acta Orthop..

[8] Hofstede S.N., Gademan M.G., Vliet Vlieland T.P., Nelissen R.G., Marang-van de Mheen P.J. (2016). Preoperative predictors for outcomes after total hip replacement in patients with osteoarthritis: A systematic review. BMC Musculoskelet. Disord..

[9] Murray D.G., Crown R.S., Dickersin K., Woods Duncan P., Epps C.H., Ettinger W.H., Friedlaender G.E., Lane J.M., Lemons J.E., Lewis J.L. (1995). Total Hip Replacement. NIH consensus conference. JAMA.

[10] Tilbury C., Schaasberg W., Plevier J.W.M., Fiocco M., Nelissen R.G.H.H., Vliet Vlieland T.P.M. (2014). Return to work after total hip and knee arthroplasty: A systematic review. Rheumatology.

[11] Horner N.S., Ekhtiari S., Simunovic N., Safran M.R., Philippon M.J., Ayeni O.R. (2017). Hip Arthroscopy in Patients Age 40 or Older: A Systematic Review. Arthroscopy.

[12] Buirs L.D., Van Beers L.W., Scholtes V.A., Pastoors T., Sprague S., Poolman R.W. (2016). Predictors of physical functioning after total hip arthroplasty: A systematic review. BMJ Open.

[13] Fransen M., McConnell S., Bell M. (2002). Therapeutic exercise for people with osteoarthritis of the hip or knee. A systematic review. J. Rheumatol..

[14] Hinton R., Moody R.L., Davis A.W., Thomas S.F., Shrier I., Feldman D.E., Gaudet M.C., Rossignol M., Zukor D., Tanzer M. (2006). Conservative no-pharmacological treatment options are not frequently used in the management of hip osteoarthritis. J. Sci. Med. Sport.

[15] Simpson D.M., Gracies J.M., Graham H.K., Miyasaki J.M., Naumann M., Russman B., Simpson L.L., So Y., Therapeutics and Technology Assessment Subcommittee of the American Academy of Neurology (2008). Assessment: Botulinum neurotoxin for the treatment of spasticity (an evidence-based review): Report of the Therapeutics and Technology Assessment Subcommittee of the American Academy of Neurology. Neurology.

[16] Pidcock F.S., Fish D.E., Johnson-Green D., Borras I., McGready J., Silberstein G.E. (2005). Hip migration percentage in children with cerebral palsy treated with botulinum toxin type A. Arch. Phys. Med. Rehabil..

[17] Eibach S., Krug H., Lobsien E., Hoffmann K.T., Kupsch A. (2011). Preoperative treatment with Botulinum Toxin A before total hip arthroplasty in a patient with tetraspasticity: Case report and review of literature. NeuroRehabilitation.

[18] Kunstcher G. (1960). Voss’ operation in OSTEOARTHRITIS. Acta Orthop. Belg..

[19] Kollberg G., Lundholm G. (1965). The Voss operation in osteoarthritis of the hip. Acta Orthop. Scand..

[20] Marchini C., Acler M., Bolognari M.A., Causero A., Volpe D., Regis D., Rizzo A., Rosa R., Eleopra R., Manganotti P. (2010). Efficacy of Botulinum Toxin type A treatment of functional impairment of degenerative hip joint: Preliminary results. J. Rehabil. Med..

[21] Macovei L.A., Rezus E. (2016). Anatomical and clinical observations on structural changes of the hip joint. Rev. Med. Chir. Soc. Med. Nat. Iasi.

[22] Gold G.E., Cicuttini F., Crema M.D., Eckstein F., Guermazi A., Kijowski R., Link T.M., Maheu E., Martel-Pelletier J., Miller C.G. (2015). OARSI Clinical Trials Recommendations: Hip imaging in clinical trials in osteoarthritis. Osteoarthr. Cartil..

[23] Rossetto O., Pirazzini M., Montecucco C. (2014). Botulinum, neurotoxins: Genetic, structural and mechanistic insights. Nature.

[24] Pirazzini M., Rossetto O., Eleopra R., Montecucco C. (2017). Botulinum Neurotoxins: Biology, Pharmacology, and Toxicology. Pharmacol. Rev..

[25] Jankovic J. (2004). Botulinum toxin in clinical practice. J. Neurol. Neurosurg. Psychiatry.

[26] Montecucco C., Schiavo G. (1995). Structure and function of tetanus and botulinum neurotoxins. Q. Rev. Biophys..

[27] Hallett M. (2000). How does botulinum toxin work?. Ann. Neurol..

[28] Kellgren J.H., Lawrence J.S. (1957). Radiological assessment of osteo-arthrosis. Ann. Rheum. Dis..

[29] Chowdhury R., Naaseri S., Lee J., Rajeswaran G. (2014). Imaging and management of greater trochanteric pain syndrome. Postgrad. Med. J..

[30] Harris W.H. (1969). Traumatic arthritis of the hip after dislocation and acetabular fractures: Treatment by mold arthroplasty. An end-result study using a new method of result evaluation. J. Bone Jt. Surg. Am..

[31] Downie W.W., Leatham P.A., Rhind V.M., Wright V., Branco J.A., Anderson J.A. (1978). Studies with pain rating scales. Ann. Rheum. Dis..

[32] Riddoch G., Rowley Bristow W., Cairns W.B., Carmichael E.A., Critchley M., Greenfield J.G., Learmonth J.R., Platt H., Seddon H.J., Symonds C.P. (1945). Medical Research Council. Memorandum No 45. Aids to the Examination of the Peripheral Nervous System.

[33] Ware J.E., Sherbourne C.D. (1992). The MOS 36-item short form health survey (SF-36). Conceptual framework and item selection. Med. Care.

[34] Perinetti G. (2016). STA-Tips Part I. Choosing statistical test when dealing with differences. South Eur. J. Orthod. Dentofac. Res..

[35] Cohen J. (1992). A power primer. Psychol. Bull..

